# Implementation and Evaluation of a Best Practice Advisory to Reduce Inequities in Technology Use for People With Type 1 Diabetes: Protocol for a Mixed Methods, Nonrandomized Controlled Trial

**DOI:** 10.2196/71038

**Published:** 2025-05-28

**Authors:** Nestoras Mathioudakis, Risa Wolf, Abha Choudhary, Georgia Davis, Mary Pat Gallagher, Meenal Gupta, Manmohan Kamboj, Nicole Rioles, Emma Ospelt, Susan Thapa, Ruth S Weinstock, Trevon Wright, Osagie Ebekozien

**Affiliations:** 1 Division of Endocrinology, Diabetes & Metabolism Department of Medicine Johns Hopkins University Baltimore, MD United States; 2 Division of Pediatric Endocrinology Department of Pediatrics Johns Hopkins University Baltimore, MD United States; 3 Division of Endocrinology Department of Pediatrics The University of Texas Southwestern Medical Center Dallas, TX United States; 4 Division of Endocrinology, Metabolism & Lipids Department of Medicine, Emory University Grady Memorial Hospital Atlanta, GA United States; 5 NYU Langone Health New York, NY United States; 6 Seattle Children's Hospital Seattle, WA United States; 7 Division of Endocrinology Department of Pediatrics at the Ohio State University Nationwide Children's Hospital Columbus, OH United States; 8 T1D Exchange Boston, MA United States; 9 SUNY Upstate Medical University Syracuse, NY United States

**Keywords:** equity, disparities, continuous glucose monitor, automated insulin delivery system, insulin pump, type 1 diabetes, best practice advisory, practice advisory, clinical decision support

## Abstract

**Background:**

Continuous advancements in diabetes technologies have improved self-management for people with type 1 diabetes. Continuous glucose monitoring and automated insulin delivery systems have enhanced the quality of life and glycemic outcomes while reducing severe hypoglycemia and diabetes ketoacidosis hospitalizations. Despite these benefits, racial inequities in the use of advanced diabetes technology (ADT) persist.

**Objective:**

This study aims to develop and evaluate a best practice advisory (BPA) within the electronic medical record (EMR) to reduce racial and ethnic disparities in ADT use. We hypothesize that an EMR-based BPA designed to standardize the prescribing of ADTs will minimize racial and ethnic disparities in ADT adoption or progression in use among pediatric and adult people with type 1 diabetes.

**Methods:**

The Best Practice Advisories to Reduce Inequities in Technology Use (BPA-TECH) study will use a nonrandomized matched pair intervention design. Phase 1 will use qualitative methods to develop and refine the BPA, including focus groups and surveys of health care providers and people with type 1 diabetes or their caregivers. Phase 2 will evaluate the effectiveness of the BPA through a controlled before-after study of people with type 1 diabetes seen at 7 T1D Exchange Quality Improvement Collaborative (T1DX-QI) centers, with control people with type 1 diabetes matched from nonintervention T1DX-QI centers. The baseline and postintervention periods will be the 12 months before and 12 months after deployment of the BPA at the intervention centers, respectively. Eligibility criteria include people with type 1 diabetes aged ≥2 years with an EMR diagnosis of T1D during the baseline period. The primary outcome is the progression in ADT use from the baseline to postintervention periods.

**Results:**

This 3-year study began in July 2024, with data collection from key stakeholders for phase 1 qualitative research beginning in August 2024. For phase 2, we estimate approximately 3000 eligible non-Hispanic Black and Hispanic people with type 1 diabetes at the intervention centers and 15,000 matched controls. Data on ADT use, glycated hemoglobin (HbA_1c_), severe hypoglycemic events, and diabetes ketoacidosis events will be collected via the T1DX-QI coordinating center. The study is powered to detect a between-group difference of 15% in the proportion of patients in the intervention and control groups in meeting the primary endpoint. We anticipate the completion of this study by May 2027.

**Conclusions:**

The BPA-TECH study aims to leverage health IT to address racial and ethnic disparities in ADT use among people with type 1 diabetes. By standardizing the approach to ADT prescribing for people with type 1 diabetes, the BPA-TECH has the potential to promote equity in diabetes management and improve clinical outcomes. The outcomes of this study will inform future efforts to reduce health care disparities.

**Trial Registration:**

ClinicalTrials.gov NCT06931275; https://clinicaltrials.gov/search?term=NCT06931275

**International Registered Report Identifier (IRRID):**

DERR1-10.2196/71038

## Introduction

Over the past decade, continuous advancements in diabetes technologies have helped to ease the burden of diabetes self-management for people with type 1 diabetes (T1D). Continuous glucose monitors (CGM) and automated insulin delivery (AID) systems have not only led to improved quality of life but also better glycemic outcomes and reduced incidence of hospitalizations for severe hypoglycemia and diabetic ketoacidosis (DKA) [1,2]. Given these clinical benefits, the American Diabetes Association now recommends that CGM be considered the standard of care for all people with T1D and that AID systems be recommended to people with T1D who are capable of safely using them [3].

Despite proven clinical benefits, racial inequities in the use of advanced diabetes technologies (ADTs), such as AID systems and CGMs, persist in both children and adults with diabetes [4-8]. Compared with Non-Hispanic White people with T1D, Non-Hispanic Black people with T1D have 1.5% higher glycated hemoglobin (HbA_1c_) levels on average [9] and are more than 3 times as likely to be hospitalized for hypoglycemia or DKA [10,11], yet they are half as likely to be using ADTs [12].

Factors contributing to these disparities include individual barriers like cost concerns, insertion pain, and alarm anxiety [13-16], as well as provider-level barriers such as insufficient knowledge, bias, and clinical decision-making criteria that do not align with current guidelines [17-19]. System-level barriers include insurance coverage issues and complicated processes for obtaining devices [20,21]. For example, a recent 7-year study at a large academic medical center found significantly lower rates of CGM discussions and use among Black people with T1D compared with their White counterparts, even after adjusting for various other factors [6]. In addition, a study by the T1D Exchange Quality Improvement Collaborative(T1DX-QI) highlighted the presence of implicit racial bias in approximately one-third of endocrinologists when prescribing ADTs [19].

Health IT (HIT) has the potential to address racial inequities in the prescribing and use of ADTs. HIT tools within the electronic medical record (EMR) include computerized provider order entry, clinical decision support (CDS) systems, patient communication portals, population health tools, and data warehouse tools among others. Recommendations for using HIT to reduce racial disparities include the standardized collection and use of race and ethnicity data, identifying inequities, tailoring quality improvement efforts, developing CDS systems for areas with significant disparities, and including input from racial and ethnic minorities in developing HIT tools [22]. CDS systems have been demonstrated to reduce racial disparities for multiple clinical conditions, including venous thromboembolism prophylaxis [23], chronic disease management [24-26], and HIV screening [27]. For example, a CDS intervention in people with heart failure led to a significant increase in referrals and guideline-recommended testing for non-Hispanic Black and Hispanic patients [28]. Compared with manual approaches, computer-based CDS systems are more effective in improving clinical practice and patient outcomes [29,30]. Evidence supports the success of CDS tools in diabetes management, where automated recommendations have led to improved glycemic outcomes and better adherence to clinical guidelines.

This Best Practice Advisories to Reduce Inequities in Technology Use (“BPA-TECH”) study will develop and evaluate a best practice advisory (BPA), an informatics alert, aimed at reducing disparities in ADT use among people with T1D. To the best of our knowledge, no CDS systems have been specifically developed or evaluated to address disparities in T1D technology use in a multicenter study. This gap is significant given the substantial evidence demonstrating the magnitude of this disparity and its adverse impact on clinical outcomes. We hypothesize that an EMR-based BPA designed to standardize the approach to people with T1D selection and prescribing of ADTs will minimize racial disparities in ADT use and progression among pediatric and adult people with T1D. We propose testing this hypothesis through a nonrandomized matched-pair intervention study, assessing a center-wide intervention among 7 clinical centers participating in the T1DX-QI.

## Methods

### Overall Study Design

**Figure 1:**
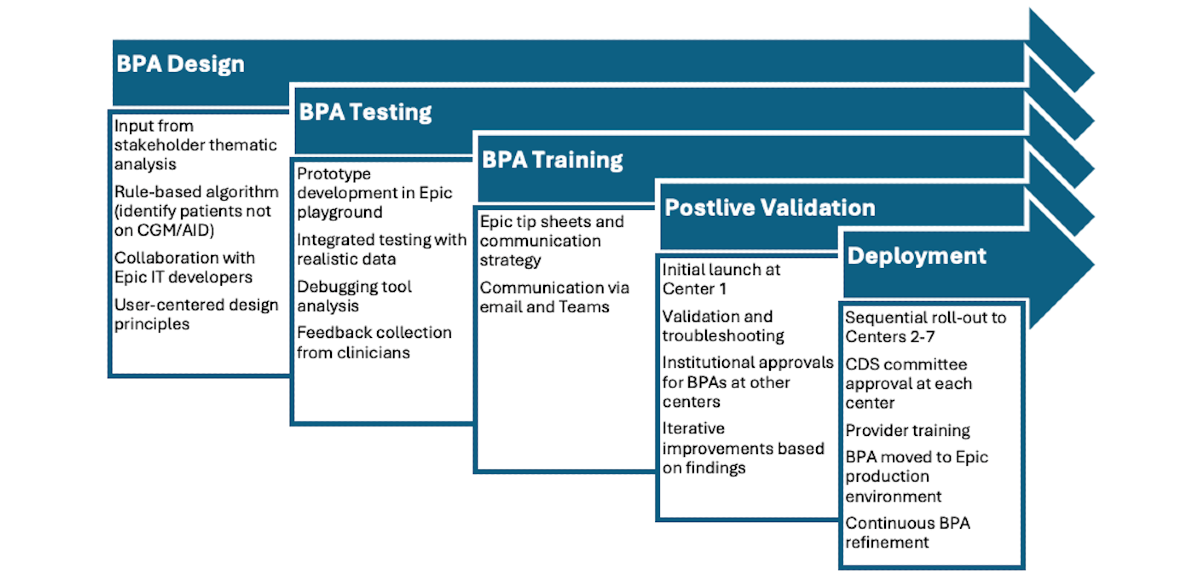
Best practice advisory development and implementation process. AID: automated insulin delivery; BPA: best practice advisory; CGM: continuous glucose monitoring.

This BPA-TECH study will use mixed methods and nonrandomized controlled trial designs (ie, controlled before-after study) to implement and evaluate a BPA aimed at reducing inequities in the use of ADTs among children and adults with T1D. Phase 1 will be a qualitative mixed methods study to develop, implement, and refine the BPA. Phase 2 will be a prospective nonrandomized matched pair intervention design to evaluate the effectiveness of the BPAs in promoting prescribing and thus patient use of ADTs. The study organizing committee will be represented jointly by principal investigators in the T1DX-QI coordinating center and Johns Hopkins University, while the T1DX-QI will be responsible for the data management and analysis. We adhered to the SPIRIT (Standard Protocol Items: Recommendations for Interventional Trials) guidelines (Multimedia Appendix 1) in the development of this study protocol and all items from the World Health Organization Trial registration dataset are included [31,32]. Protocol amendments will be communicated during monthly meetings between the organizing committee and the participating clinical sites, and updates will be posted on ClinicalTrials.gov. The timeline for this 3-year study is shown in Figure 1.

### Study Setting

In 2016, through financial support from the Leona M. and Harry B. Helmsley Charitable Trust, T1D Exchange established the T1DX-QI, which initially included 10 diabetes and endocrinology centers focused on population health by advancing quality improvement, health equity, and real-world studies. The network has since expanded to include 62 T1D and 20 type 2 diabetes (T2D) centers, along with partners in primary care.

T1DX-QI manages more than 10 multicenter projects annually, with 4-10 centers participating per project, and has expanded its scope to include equitable access to diabetes technology and devices for both T1D and T2D populations.

In phase 1 of the study, we will recruit health care providers specializing in diabetes management from academic hospitals affiliated with the T1DX-QI. People with T1D or their caregivers will be recruited from the T1DX online registry, which includes individuals across the United States who may receive care in community clinics or academic hospitals. Phase 2 of the study will also encompass participating clinical sites affiliated with the T1DX-QI, specifically academic medical centers.

### Phase 1: Implementation

#### Stakeholder Input

During the qualitative phase of this study, we will use electronic surveys and conduct focus group sessions and structured interviews of (1) pediatric and adult endocrinology and diabetes health care providers from centers who are part of the T1DX-QI and (2) people with T1D and their caregivers. The objective of the qualitative phase of this research study is to solicit stakeholder input regarding the perceived utility, design, functionality, benefits, and potential challenges of a BPA targeting increased ADT prescribing for people with T1D. We will use the Agency for Healthcare Research and Quality (AHRQ) “Five Rights” for effective CDS [33] (Table 1) and the T1D Exchange Health Equity Framework as guiding frameworks [34].

For the health care providers, 8 focus groups will be conducted with a multidisciplinary group of providers (approximately 3 to 5 participants per focus group) selected from centers participating in the T1DX-QI collaborative to understand systems barriers to incorporating a BPA, criteria that would trigger the BPA, and reasons for deciding not to prescribe ADTs to patients. The focus group guide is provided in Multimedia Appendix 2. Eligible participants will be physicians, advanced practice providers, certified diabetes care and education specialists, and nurses who are involved in the care of patients with T1D and have expertise in the management of ADTs along with IT specialists involved in the development of BPAs. An incentive of US $150 remuneration will be provided to focus group participants. In addition, 4 questions related to the proposed BPA were sent to all participating centers in the T1DX-QI as part of their annual survey (Multimedia Appendix 3).

**Table 1:** Agency for Health Care Research and Quality 5 rights of effective clinical decision support frameworks to guide electronic surveys and focus group sessions.

Five rights of clinical decision support	Provider	Patients
Right information	How to translate ADA^a^ standards of care for CGM^b^ and insulin pumps into prompt and alert? Reason device not offered or declined?	What information would be helpful to you in making a decision about using ADT^c^? Reasons device declined?
Right person	Who should receive the prompt? Endocrinologist or diabetes advanced practice provider? Primary care physician?	Who should receive the information about technology (patient only, parent, primary care physician)?
Right intervention format	Define BPA^d^ inputs	MyChart notification?
Right channel	Order entry Progress note template Level of service (closing chart) Health maintenance or care gap	MyChart notification Previsit questionnaire Postvisit questionnaire
Right timing	Previsit charting During encounter When closing encounter How often? Once, 3 months, 6 months, 12 months?	Before visit? After visit? How often? Once, 3 months, 6 months, 12 months?

^a^ADA: American Diabetes Association.

^b^CGM: continuous glucose monitoring.

^c^ADT: advanced diabetes technology.

^d^BPA: best practice advisory.

All participating intervention centers currently use the Epic EMR system; therefore, focus group sessions will also be guided by Epic’s CDS setup and support guide as a reference to inform the questions for the focus group session and qualitative interviews. It is important to note that EHR systems often use varying terminology to describe CDS alerts. For instance, the Epic EMR vendor recently renamed its “Best Practice Advisory” to “Our Practice Advisory.” To maintain consistency throughout this study, we will use the term “BPA” to refer to CDS alerts.

For people with T1D and caregivers, we will administer a 20-item Qualtrics questionnaire on the T1D Exchange Online Registry, which is accessed by approximately 19,000 caregivers or individuals with T1D who have consented to be contacted about additional studies. People with T1D or caregiver survey (Multimedia Appendix 4) will address (1) the perceived need for the BPA, (2) benefits and concerns, (3) relevant information and communication approaches, (4) the importance of person-centered care, and (5) user interface and alerts. The goal is to survey approximately 50 caregivers of youth with T1D and 50 adults with T1D. Participants completing the electronic survey will be offered the opportunity to participate in a follow-up structured interview.

We will conduct structured interviews with approximately 10 caregivers of youth with T1D and 10 adults with T1D to capture people with T1D perspectives on BPAs related to ADTs (Multimedia Appendix 5). For both the interviews and survey, we will recruit a representative sample of 50% non-Hispanic Black and Hispanic people with T1D. Participants will receive a US $100 payment for time remuneration.

All focus group sessions for both health care providers and people with T1D or their caregivers will be conducted by T1DX-QI staff trained in qualitative interviewing. Focus group sessions will be audio-recorded, transcribed, and exported into NVivo software (NVivo version 13, Lumivero) for data organization and management and will continue until thematic saturation is reached. Focus group guides may be iteratively adapted based on insights gathered during the sessions.

#### Intervention Centers

A total of 7 T1DX-QI clinical centers, consisting of pediatric and adult diabetes specialty clinics in the United States (Table 2), will participate in both the development and implementation of the BPA. A 7-item Qualtrics survey was distributed to all clinical centers within T1DX-QI to gather information on their EMR systems, capacity to develop automated alerts for CGMs and AIDs, IT capacity and commitment to the project, anticipated EMR freezes or delays during the planned intervention period, typical timeline for EMR changes at the institution, and previous experience building BPAs. Based on these responses, centers were selected to ensure geographical representation of the United States and a balance of pediatric and adult centers, with priority given to clinical centers using Epic EMR and demonstrating capacity, interest, and commitment to the project.

**Table 2:** Intervention clinical centers.

Intervention center	Clinical center	Geographical region	Pediatric or adult
1	Johns Hopkins	Mid-Atlantic	Pediatric and adult
2	Hassenfeld Children’s NYU^a^	Northeast	Pediatric
3	Upstate Medical University	Northeast	Adult
4	Nationwide Children’s	Midwest	Pediatric
5	Grady Memorial	Southeast	Adult
6	University of Texas Southwestern	Southwest	Pediatric
7	Seattle Children’s Hospital	Northwest	Pediatric

^a^NYU: New York University.

Each center will have a designated principal investigator who will participate in monthly meetings with the organizing committee. Johns Hopkins University, which includes both pediatric and adult diabetes centers, will be the primary center for the development of the BPA. This center will oversee prototype development, testing, and postlive validation.

#### BPA Development

The BPA development phase of this study will encompass several stages: design, testing, training, postlive validation, deployment, and ongoing refinement (Figure 1).

##### BPA Design

Using a rule-based algorithm, the EMR-based BPA will be designed to recommend ADT prescriptions for people with T1D not already using an AID system, and this would also encompass an alert for people not using CGM (and thus not using AID). The specific functionality of the BPA will be guided by the thematic analysis of the stakeholder input obtained in phase 1. The function will generate a BPA if a patient is not using an AID system and will suggest to the provider to discuss and prescribe technologies for using an AID system.

The BPA design will be led by the study organizing committee with direct involvement and support from Epic IT analysts and developers at Johns Hopkins University. Iterative feedback will be obtained from the principal investigators at each of the participating intervention centers during the prototype development. Table 3 outlines the key considerations for the design team. Throughout the design process, Norman’s user-centered design principles will be applied to ensure the BPA is intuitive, user-friendly, and meets the needs of all stakeholders [35].

**Table 3:** Key considerations for best practice advisory (BPA) design team.

Consideration	Descriptions
Stakeholder input	Incorporate thematic analysis of feedback from patients, caregivers, and health care providers.
Algorithm rules	Define criteria for triggering the BPA based on patient EMR^a^ data.
Workflow integration	Ensure BPA integrates seamlessly into the provider’s workflow.
Iterative testing	Conduct iterative testing and refinement based on feedback from pilot implementations.
Setting and restrictions	Consider the setting (ambulatory, inpatient, both) and specific locations such as endocrinology clinics, primary care clinics, or health-system wide.
Location in Epic	Determine the BPA’s location in Epic, such as Windows, BPA Navigator, or Flag in Storyboard.
Active versus passive	Decide between active (windows that appear in the user’s Epic workflow) and passive (appear within assigned locations that fit into the end user’s workflow) BPAs.
Clinician action	Specify actions clinicians can take, such as placing orders or referrals.
Triggering messages	Determine language in the BPA, including potential references to practice guidelines, explain the reason for the BPA, and suggest actions to be taken.
Iterative testing	Conduct iterative testing and refinement based on feedback from pilot implementations.

^a^EMR: electronic medical record.

##### BPA Testing

After a prototype BPA is developed, the testing phase will involve incorporating the BPA into integrated testing scripts in the Epic sandbox environment. The prototype BPA will be tested thoroughly in an environment using realistic data. A debugging tool will test specific cases in the BPA builder before actual deployment. Site principal investigators will be asked to provide direct oral or written feedback about the usability of the BPA and will be encouraged to suggest improvements.

##### BPA Training

Minimal provider training is expected as BPAs are widely used in the EMR. However, Epic tip sheets and a communication strategy will be developed by each of the intervention centers to facilitate the training process. Communication methods may include email and Microsoft Teams posts to ensure that all relevant staff are informed and prepared to use the BPA effectively.

##### Postlive Validation

Initially, the BPA will be launched at Center 1 (Johns Hopkins University) to allow for an in-depth assessment and troubleshooting of any potential issues before wider deployment. This initial launch will involve a thorough 90-day validation phase, during which the functionality and impact of the BPA will be closely monitored and evaluated. During this validation phase, any issues or inefficiencies identified will be addressed and rectified. In addition, during this period, the intervention centers will begin the process of obtaining local institutional approval for the BPA. This approach ensures that the BPA is fully optimized before it is implemented at the remaining 6 intervention centers (centers 2-7).

##### Deployment

**Figure 2:**
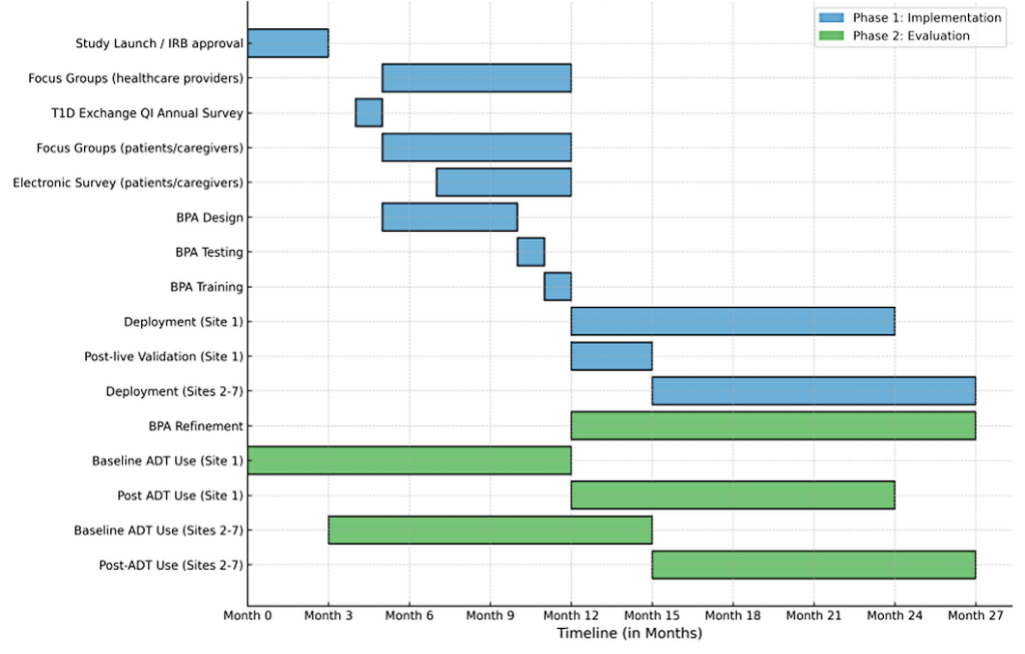
Study timeline. ADT: advanced diabetes technology; BPA: best practice advisory; IRBL institutional review board; QI: quality improvement; T1D: type 1 diabetes.

Following the successful completion of the validation phase at Johns Hopkins University, the BPA will be rolled out sequentially to the other intervention centers. Each institution has a CDS committee that approves new builds, and this approval process will be factored into the timeline (Figure 2). Each of the participating intervention centers has already received letters of support from institutional IT leadership; therefore, we do not anticipate any approval barriers. To ensure that each intervention center is prepared to launch the BPA at the same time, the organizing committee will recommend that the approval process for the build occur at least 90 days before the planned launch. This staged deployment strategy minimizes the risk of widespread issues and allows for iterative improvements based on real-world feedback. Once the study team has ensured that the target providers at the other 6 participating centers have received requisite training, the BPA will be moved into the Epic production environment, which will correspond to the start of the intervention phase of the study.

After deployment, we will monitor adherence to the BPA using SlicerDicer, an analytics tool integrated within Epic. This tool enables us to track the frequency of BPA activations, the acknowledgment reasons selected by clinicians, and the subsequent actions taken. SlicerDicer allows for the creation of custom reports and dashboards that visualize compliance trends over time and facilitates in-depth analysis of specific instances of noncompliance. These insights will be used to generate feedback reports for clinicians, enabling clinical leaders to compare provider compliance rates against their peers and internal benchmarks. This approach aims to encourage adherence to BPAs and foster a culture of continuous quality improvement.

##### Refinement

Feedback from the providers at the 7 participating centers will be collected following the implementation of the BPA and periodically during the 12-month assessment period. This feedback will be used to refine the BPA in an iterative fashion.

### Phase 2: Evaluation (Study Design)

Using a nonrandomized matched pair-intervention design (ie, controlled before-after study), we will compare the use of ADT following the BPA intervention among non-Hispanic Black and Hispanic people with T1D at 7 participating T1DX-QI centers. These will be matched with control non-Hispanic Black and Hispanic people with T1D at nonintervention T1DX-QI centers over a 12-month period. Both control and intervention centers have completed the EMR data mapping to the T1DX-QI, allowing for consistent outcome assessment across both groups. The study will consist of 2 distinct periods:

1. Baseline period (before): 12 months before deployment of the BPA at the intervention centers.
2. Implementation period (after): 12 months after the deployment of the BPA at the intervention centers.

The before-after matched-pair intervention design allows for a real-world assessment of the BPA’s impact on ADT use while controlling for key confounders. Comparing ADT prescribing before and after BPA implementation within intervention sites and using matched controls from nonintervention centers, the design also accounts for site-specific prescribing patterns and secular trends. This quasi-experimental approach is practical for an EMR-based intervention and minimizes selection bias by matching key clinical and demographic variables.

### Eligibility Criteria

Inclusion and exclusion criteria are listed in Textbox 1.

**Inclusion and exclusion criteria.**

**Inclusion criteria**

- Age ≥2 years
- A diagnosis of type 1 diabetes (determined using *ICD-10* [*International Statistical Classification of Diseases and Related Health Problems, Tenth Revision*] diagnosis codes [E10.X] and other various discrete data elements, such as flowsheet rows, as mapped by clinical centers to the Type 1 Diabetes Exchange Quality Improvement Collaborative database).
- Receiving care at one of the seven intervention centers or a matched control center in the Type 1 Diabetes Exchange Quality Improvement Collaborative.
- Patients with at least one clinical encounter (in-person or video visit) in both the baseline and follow-up periods.
- Not prescribed an automated insulin delivery system (patients prescribed only continuous glucose monitoring are eligible, provided they are not prescribed an automated insulin delivery system).

**Exclusion criteria**

- People with type 1 diabetes already using an automated insulin delivery at baseline.
- Pregnant patients.
- People with type 1 diabetes receiving end-stage kidney disease dialysis.
- People with type 1 diabetes using hydroxyurea.

### Intervention Group

People with T1D meeting the eligibility criteria at one of the 7 intervention centers where the BPAs are deployed will be assigned to the intervention group. We estimate that there are approximately 11,496 eligible people with T1D in the intervention centers.

### Control Group

Matched control people with T1D will be selected from the pool of nonintervention clinical centers sharing EMR data with the T1DX-QI coordinating center with over 80,000 people with T1D data available in the database.

To accurately match people with T1D between the intervention and control groups, we will identify and include a comprehensive set of covariates that may influence the likelihood of receiving the BPA. These covariates will include both patient-level and clinical center-level characteristics. Patient-level characteristics will include demographics (age, sex, race, and ethnicity), socioeconomic status (insurance type and zip code–based income), and clinical characteristics (duration of diabetes, comorbidities, HbA_1c_ levels, etc). Clinical center-level characteristics will include clinic size and resources (number of people with T1D seen annually, number and type of clinical full-time equivalents, geographic location, patient population demographics, and historical usage rates of CGMs and AID systems. Intervention and control groups will be matched using the propensity score method.

Before propensity score matching, any missing baseline covariate data will be imputed using predictive modeling techniques. Multiple imputation by chained equations will be used to generate multiple complete datasets, which will then be averaged to create a single dataset for the matching process. This approach ensures that the imputed values are based on observed data, maintaining the integrity and representativeness of the dataset.

Propensity scores will be estimated using logistic regression, where the outcome variable is the likelihood of receiving the BPA intervention. The model will include the identified patient-level and clinical center–level covariates as predictors. The estimated propensity score represents the predicted probability that a given patient would receive the BPA based on their covariate profile. Patients in the intervention group will be matched to patients in the control group using a 1:4 matching ratio. Nearest neighbor matching without replacement will be used, ensuring that each control patient is only matched once. The matching process will aim to minimize the distance between propensity scores of matched pairs, thus creating comparable groups. The quality of the matching process will be assessed by comparing the distribution of covariates between the matched intervention and control groups. Standardized mean differences will be calculated for each covariate to ensure balance, with a standardized mean difference of less than 0.1 indicating adequate balance. In addition, a visual inspection of propensity score distributions before and after matching will be conducted to confirm the success of the matching process.

To assess the robustness of the results, sensitivity analyses will be performed using different matching algorithms (eg, caliper matching and kernel matching) and varying the matching ratio (eg, 1:3). In addition, the impact of imputation on the results will be examined by comparing outcomes using datasets with and without imputed values.

For nonstatic matching variables (eg, laboratory results), the last available result in the baseline period of 12 months before the BPA deployment date in the intervention centers will be used.

### Primary Outcome

The primary outcome will be progression in ADT use (as documented in the EMR) during the 12-month study period. For both intervention and control centers, a participant’s baseline ADT status will be ascertained in the 12-month period before deployment of the BPA intervention. The primary outcome will be defined as positive for an individual if any of the following transitions from the baseline period to the implementation period occur with respect to ADT:

1. No CGM → CGM.
2. No AID → AID.

The T1DX-QI database includes variables that will be used to evaluate the study outcome (eg, medication list and flowsheet data). We have extensively published on the T1DX-QI data source and its validation, and have used the database for several real-world studies [36-42].

### Adverse Events

It is not anticipated that the intervention will pose any additional risk of harm to people with T1D, particularly given the established benefits of AIDs and CGMs in enhancing glycemic control and reducing the risk of hypoglycemia. Nevertheless, the study will closely monitor rates of serious hypoglycemic events and DKA events, using existing data mapped from control and intervention sites to the T1DX-QI database. These rates will be compared between the intervention and control sites to ensure that there are no unexpected or unintended consequences of the intervention on adverse glycemic outcomes.

### Statistical Analysis

#### Data Collection

Currently, more than 102,000 people with T1D have EMR data in the T1DX-QI database. Mapped outcome data include ADT use, HbA_1c_, severe hypoglycemic events, and DKA events, and are automatically transmitted to the T1DX-QI database monthly. Data from all intervention and control sites will be analyzed by the T1DX-QI data coordinating center. All data are anonymized and deidentified to ensure patient privacy. Protected health information is in the form of a limited dataset, which includes dates but excludes any direct identifiers. Medical record numbers are never shared with T1DX. Unique identifiers are created by the hospitals and shared with T1DX for the purpose of longitudinal (encounter-level) data analysis. Results are aggregated, and no encounter-level data are published, further safeguarding participant confidentiality. The data monitoring committee will be composed of the principal investigators from JHU and T1DX-QI, along with biostatisticians, and will operate independently from the sponsor. Members of the data monitoring committee will have access to the final trial dataset.

#### Power

This study is powered to detect a between-group difference of 15% in the proportion of patients in the intervention and control groups meeting the primary endpoint. The matched-pair control assessment of 15% progression in ADT use is estimated from T1DX-QI data over a previous 12-month period. The largest sample size necessary for 90% power if the absolute difference in the proportion of ADT progression is only 5% (relative increase of 25%) is 589 matched pairs. If possible, we will attempt to match intervention to control patients in a 1:2 fashion with 2 matched controls per intervention patient. An interim analysis of the intervention group event rate may be conducted to inform whether additional matched pairs are needed.

### Analysis

#### Phase 1

For survey data, Likert scale responses will be coded numerically for qualitative analysis. Descriptive statistics will be calculated to summarize the data, and differences between groups (ie, based on demographic variables) will be explored. For the structured interviews and focus groups, a thematic approach will be used to identify, analyze, and report patterns with the qualitative data. NVivo software will be used, which provides features for coding, organizing, and visualizing data [43]. Both deductive and inductive coding methodologies will be used to link similar codes to broader themes based on significant phrases, concepts, and ideas present in the transcripts [44]. Themes will be reviewed and refined through discussions among the research team to ensure consistency and reliability.

#### Phase 2

The primary outcome will evaluate the impact of the BPA intervention on patients’ progression of ADT use using a generalized linear mixed model to account for matched pairs and random center effects. The model will be adjusted for the number of encounters and other possible confounders not already matched.

The secondary outcome analyses will compare differences in ADT use between non-White and White people with T1D in the intervention and control groups. Additional secondary and exploratory outcomes will be assessed using the appropriate statistical model for the outcome adjusting for patient- and center-level confounders. Exploratory analysis assessing pediatric and adult centers separately will be performed.

In cases where ADT is not prescribed by the provider, we will explore the reasons why, and why the diabetes technology was not offered or accepted by patients. We will quantify the BPA responses as provider-led or patient-led reasons for not advancing ADT use. We will perform multivariable logistic regression analysis to evaluate whether patient characteristics (demographics, race, ethnicity, diabetes characteristics, and complications) are associated with the reasons that technologies are not offered or declined.

### Ethical Considerations

This intervention study was registered on ClinicalTrials.gov (NCT06931275) and received approval from the WCG institutional review board on April 26, 2024 (institutional review board approval number 45614667). For the qualitative research proposed in phase 1 of the study, oral informed consent will be obtained from all participants (patients, caregivers, and health care providers) for the focus groups and interviews, and written informed consent will be obtained from all participants (patients, caregivers, and health care providers) for the surveys. However, due to the nature of this implementation study, informed consent cannot be obtained from participants during the observation phase (phase 2), which will evaluate the impact of a before-and-after design on clinical outcomes.

## Results

The BPA-TECH study funding was finalized in April 2024. Data collection for the phase 1 qualitative component of this study began in August 2024 and is expected to be completed around July 2025. Phase 2 will commence with the deployment of the BPA, followed by a 12-month evaluation period that is expected to conclude around July 2026. We estimate evaluating data from 3000 eligible people with T1D across the 7 clinical centers and 15,000 possible matched controls across 41 centers.

## Discussion

### Principal Findings

The overarching goal of this study will be to promote the use of ADT and enhance health equity in ADT use for diabetes management in people with T1D. While previous research has illustrated the many barriers and racial disparities in ADT use, the focus of this initiative is to develop a comprehensive and evidence-based solution using HIT to address and mitigate ongoing disparities in ADT use and outcomes. We expect that clinical centers implementing the BPA will demonstrate higher rates of progression to ADT use compared with matched controlled sites, as well as a smaller gap in the use of these technologies between White and non-White patients.

In a 2-phased approach, this protocol aims to develop a BPA with input from key stakeholders, and then evaluate the effectiveness of the BPA in reducing disparities in ADT use among people with T1D. The goal of this intervention is to increase the progression of ADT use overall, while reducing health disparities and improving outcomes in all people with T1D. In addition, reasons for not prescribing ADT in response to the BPA will be collected. If successful, the build for this BPA can be disseminated to other hospital systems for implementation to improve outcomes on a larger scale.

To our knowledge, this is one of the first studies to develop and use a CDS initiative in the EMR to promote equitable discussion and prescribing of ADT in people with T1D. In addition, to increase the generalizability of our findings, a diverse sample of People with T1D will be included from multiple centers across the United States to account for geographical and systems-level (ie, state-level and insurance-based) variabilities.

Previous initiatives to improve the use of diabetes technologies in people with T1D have focused on patient-level interventions. Early initiation of CGM soon after diagnosis of T1D, and point-of-care CGM placement and education during routine diabetes care visits have led to increased uptake of CGM and improved glycemic outcomes [45-47]. A current randomized control trial deploying a diabetes navigator will determine if the help of a technology liaison can improve uptake and sustained use of ADT in people with T1D [48].

The use of a BPA as a CDS tool in this protocol serves as an intervention at both the provider and system level, enabling greater scalability to create lasting improvements in ADT uptake and glycemic outcomes. There is previous evidence supporting the successful implementation of computer-based decision support in diabetes care [49-51]. One cluster randomized clinical trial consisting of 14 primary care centers (66 primary care physicians and 697 patients with T2D on insulin therapy) in Madrid, Spain successfully implemented a computer application designed to help primary care physicians make decisions about insulin therapy [52]. The design of the algorithm included the recommendation to change the insulin dose and the insulin regimen when necessary. The people receiving care at the 7 centers randomly assigned to use the algorithm had a significant reduction in HbA_1c_ compared with the people at the 7 centers in the control group [52]. Another meta-analysis of 70 randomized controlled trials assessing the critical features of CDS systems that improve clinical practice highlighted 4 initiatives strongly associated with a successful center decision support system: (1) CDS provided automatically as part of clinical workflow, (2) CDS delivered at the time and location of decision-making, (3) actionable recommendations provided such as prescriptions, and (4) computer-based [53]. In this protocol, the BPA is designed to incorporate these 4 critical features and thus we expect to see improvement in ADT uptake at the intervention sites.

This study leverages the T1DX-QI consortium, which has a robust quality improvement and health equity framework to evaluate the impact of an EMR-based BPA on ADT use and racial disparities. By implementing the intervention at 7 motivated sites with strong research and QI expertise, and strong IT infrastructure, the study ensures effective troubleshooting and optimization of the BPA. This real-world, multicenter approach enhances generalizability and supports the potential for scalability to other centers if successful. In addition, integrating health equity principles strengthens the study’s ability to assess disparities and inform broader systemic improvements in diabetes care.

Although this study is limited to centers using the Epic EMR system to streamline the development of the BPAs, the BPA prototypes developed in this study could be adapted for other EMR systems in the future. Further, this study is limited to centers participating in the T1DX-QI, where there is likely to be greater motivation to improve outcomes and close care gaps, and thus findings may not be generalizable to all centers providing diabetes care. However, the use of BPAs allows standardization of practice and can serve as an equalizer across different practices.

### Dissemination of Findings

The findings will be published in diabetes, health technology, and health equity–focused medical journals, and will be presented at major national and international conferences. Patient-friendly summaries will be developed and shared with patients and caregivers, while educational materials and training programs will be created for health care providers to facilitate clinical implementation on a larger scale. Authorship eligibility for publications resulting from this study will adhere to the International Committee of Medical Journal Editors (ICMJE) guidelines [54].

### Conclusions

The research approach outlined in this protocol aims to leverage health IT to reduce racial disparities in prescribing and use of ADTs, thereby promoting equity, improving disease management, and enhancing outcomes in people with T1D.

## Appendix

Multimedia Appendix 1 SPIRIT checklist.

Multimedia Appendix 2 Focus group session guide for health care providers.

Multimedia Appendix 3 2024 Type 1 Diabetes Exchange Quality Improvement Collaborative Annual Center Survey questions related to the proposed best practice advisory.

Multimedia Appendix 4 Electronic survey for people with type 1 diabetes and their caregivers.

Multimedia Appendix 5 Focus group session guide for people with type 1 diabetes and their caregivers.

Multimedia Appendix 6 ChatGPT prompts used to generate figures.

## Abbreviations

ADT: advanced diabetes technology
AHRQ: Agency for Healthcare Research and Quality
AID: automated insulin delivery
BPA: best practice advisory
BPA-TECH: Best Practice Advisories to Reduce Inequities in Technology Use
CDS: clinical decision support
CGM: continuous glucose monitoring
DKA: diabetic ketoacidosis
EMR: electronic medical record
HbA_1c_: glycated hemoglobin
HIT: health IT
SPIRIT: Standard Protocol Items: Recommendations for Interventional Trials
T1D: type 1 diabetes
T2D: type 2 diabetes
T1DX-QI: Type 1 Diabetes Exchange Quality Improvement Collaborative

## Data Availability

Data sharing is not applicable to this article as no datasets were generated or analyzed during this study.
