# Supplementary material for: Implementation and Evaluation of a Best Practice Advisory to Reduce Inequities in Technology Use for People With Type 1 Diabetes: Protocol for a Mixed Methods, Nonrandomized Controlled Trial
Source: JMIR Res Protoc. 2025 May 28;14:e71038. doi: 10.2196/71038 (PMC12159554; doi:10.2196/71038)
Supplement: Multimedia Appendix 2 [file resprot_v14i1e71038_app2.docx]

**Focus Group Guide for Providers Regarding Best Practice Advisory** (**BPA) Development**

**Introduction**

*Thank you for your time and willingness to participate in this focus group. My name is [facilitator name] and I work with the T1D Exchange as a senior data analyst and I will be facilitating this focus group today. My colleague Trevon is joining us as well and he is acting project manager for this study.*

*Before we begin, I would like to give you a little more information about the purpose of this research. This study is being conducted by T1D Exchange. This study is sponsored by Breakthrough T1D.*

*We invited diabetes-related healthcare providers and care team members with real world experience prescribing and educating patients for advanced diabetes technologies to discuss best practice advisories (BPA) and their design, use, and triggers. The goal of this project is to create a BPA to standardize prescribing of CGMs and Automatic Insulin Delivery Systems (AID)s systems for children and adults with type 1 diabetes.*

*This focus group will last approximately 60 minutes and the audio will be recorded for note taking purposes. This recording will be destroyed after the study is completed. Names and personally identifiable information will not be reported.*

***You will receive a 150 dollar giftcard*** *for your time today*

*Do you have any questions or concerns before we begin? <****Start recording*** *>*

**Participant Background Information**

- Please share your current role on your team & how many years have you been in practice? (0-5 years, 6-10 years, 10+ years)
- Do you prescribe diabetes-related devices such as CGM and insulin pumps, smart insulin pens to patients? OR do you provide training/education for diabetes-related devices OR both?

**General Feelings and Need for Best Practice Advisory**

- How effective do you find Best Practice Advisory’s (BPA) to be in your practice?
  - Strengths & weaknesses
- Which diabetes-related technologies should BPAs be implemented for?
- Do you think a BPA would be useful for prescribing CGMs?
- Do you think a BPA would be useful for prescribing automated insulin delivery (AID) systems for patients with type 1 diabetes?
  - Both CGM and AID/ one or the other
  - Why or why not?
- Does your center have existing BPAs in your EHR related to diabetes technologies?
  - If so, what does your current process look like?
- Considering your current process, what do you think should be considered when developing/ implementing a BPA?

**BPA Design and Implementation Workflow**

- What information or guidance would be most helpful in a BPA for advanced diabetes technologies? (what would you like to see prompted on the screen)
- Please describe the key features you think should be included in a BPA for CGMs.
- Please describe the key features you think should be included in a BPA for AID.
  - Any additional features included for one that is not in the other?
- Walk me through how a BPA for diabetes related technology (CGM or AID) may be integrated into the existing workflow to minimize disruption?
  - What staff may be involved?
  - What resources would this require?
  - Other logistical considerations?
- Where in the Epic EMR should the BPA be configured? Why?
  - Examples (Storyboard, BPA Navigator, Pop-up Window)
- What are potential barriers to adoption of a BPA for advanced diabetes technologies in your practice?
  - - Clinic-specific
    - Patient-specific
    - Provider-specific

**Clinical Decision Support**

- What criteria should trigger the BPA for CGMs?
- What criteria should trigger the BPA for AIDs?
  - Example of criteria in general (question probes)
    - Level of diabetes glycemic control
    - Based on devices patient is already using
    - Stepwise firing of multiple devices
    - Frequency of BPA

**Appearance and Frequency of BPA**

- What should cause the BPA to appear or no longer appear?
- How often should the BPA appear to the clinician? (for a patient level)
  - Every visit? Annually?
  - Difference for CGM/AID
  - Triggered only during synchronous clinical encounters (i.e. office visit, telemedicine) and not during asynchronous work (i.e. chart review, phone call, response to PwT1D message)
  - Restricted to fire only in the ambulatory/outpatient setting (vs. ED/inpatient)
- Should the BPA be active or passive?
  - Active means that windows appear in the user’s workflow.
  - Passive means they are assigned locations that fit into the user’s workflow.

**Target Audiences and Preferences**

- Considering the **workflow** for a BPA- How does your clinical team feel about the following processes?
  - Who should the BPA be targeted to on your care team?
    - Targeted to each member of the diabetes/endocrine team
    - Targeted to primary care provider
  - Please share your opinion on your preference of stopping or postponing of BPAs
    - A hard stop (cannot close clinical encounter without addressing BPA)
    - A soft recommendation (appears in health maintenance or care gaps section of EMR as prompt)
  - If too busy to address or competing priorities do you feel a BPA should be able to be postponed to a future encounter?
    - Able to be suppressed for a defined period of time (e.g. 6 months, 1 year) for the *healthcare team member* who responded to the BPA
    - Able to be suppressed for a defined period of time (e.g. 6 months, 1 year) for the *entire diabetes care team* if any member responded to the BPA
- How can the BPA be designed to support clinical decision-making without causing alert fatigue?

**Direct Actions**

- What direct action should clinicians be able to take from the BPA?
- How important is it that the BPA takes you to an order panel where CGMs/AIDs can be prescribed directly?
- If a patient declines, how important is it for automated information to be provided in after visit summary (including resources)
- If a BPA fires incorrectly-what should happen to guide the provider…
  - Example- Patient has CGM (AID), but is not in system
  - Example- Patient is not using CGM (AID) anymore but still in system

**Benefits of BPA**

- - What are some potential benefits of using a best practice advisory for advanced diabetes technologies?
    - For patients?
    - For the clinic/workflow?
    - For tracking/measuring
