# Supplementary material for: Implementation and Evaluation of a Best Practice Advisory to Reduce Inequities in Technology Use for People With Type 1 Diabetes: Protocol for a Mixed Methods, Nonrandomized Controlled Trial
Source: JMIR Res Protoc. 2025 May 28;14:e71038. doi: 10.2196/71038 (PMC12159554; doi:10.2196/71038)
Supplement: Multimedia Appendix 3 [file resprot_v14i1e71038_app3.pdf]

Best Practice Advisory

. Best Practice Advisory

. We are planning a best practice advisory (BPA) to standardize prescribing of CGM and insulin pump technologies for children and adults living with type 1 diabetes. The hypothesis is that by standardizing the recommendation for prescribing these technologies, it may be a potential strategy for addressing racial/ethnic inequities in technology use. We would like your center's opinions about BPAs in general and their potential application to address this problem. For the questions below, insulin pump refers to automated insulin delivery systems (hybrid closed loop pumps).

Q27. At your center, do you have any existing BPAs related to diabetes technologies?

- Yes
- No
- Unsure/Unknown

Q28. For the PWD at your center, how useful do you think a BPA would be in

	Strongly Disagree	Disagree	Neither Disagree or Agree	Agree	Strongly Agree
Reminding you to discuss CGM	○	○	○	○	○
Reminding you to discuss insulin pump	○	○	○	○	○
Make you more likely to prescribe a CGM	○	○	○	○	○
Make you more likely to prescribe an insulin pump	○	○	○	○	○
Increasing overall uptake/use of CGM by your patients	○	○	○	○	○
Increasing overall uptake/use of or insulin pump by your patients	○	○	○	○	○
Reducing racial/ethnic inequities in prescribing of CGM	○	○	○	○	○
Reducing racial/ethnic inequities in prescribing of insulin pumps	○	○	○	○	○

Q29. For the PWD at your center, who is the best person on the healthcare team for the BPA to target? Rank in order (1= most appropriate to 4= least appropriate):

Prescribing clinician (MD/DO, Advanced Practice Provider [NP,PA])

Clinic nurse

Certified diabetes care and education specialist

Other [ ]

Q30. Does your center routinely offer Automated Insulin Delivery (AID) within 6 months of diagnosis?

- Yes
- No
- Unsure/Unknown

T1DX-QI Experience

. **T1DX-QI Experience**

Q31. Please select your level of satisfaction with being involved in the T1DX-QI
