# Supplementary material for: Implementation and Evaluation of a Best Practice Advisory to Reduce Inequities in Technology Use for People With Type 1 Diabetes: Protocol for a Mixed Methods, Nonrandomized Controlled Trial
Source: JMIR Res Protoc. 2025 May 28;14:e71038. doi: 10.2196/71038 (PMC12159554; doi:10.2196/71038)
Supplement: Multimedia Appendix 4 [file resprot_v14i1e71038_app4.docx]

Caregiver

Thank you for participating in this survey. Your feedback will help us develop a Best Practice Advisory (BPA) to standardize prescribing Continuous Glucose Monitors (CGMs) and Automated Insulin Delivery systems (AIDs) for children and adults with type 1 diabetes.

A Best Practice Advisory (BPA) is a type of clinical decision support tool used in electronic health records. BPAs provide alerts and reminders to healthcare providers to ensure that they are following the latest medical guidelines and standards. Current clinical practice guidelines recommend CGMs for all patients with type 1 diabetes, and AIDs are strongly recommended for patients who can safely use them. Examples of CGMs include Dexcom G7 and Freestyle Libre 3. Examples of AIDs include Tandem Control IQ, Omnipod 5, Medtronic 780G, and iLet Bionic Pancreas.

Despite the benefits of CGMs and AIDs, there are significant racial gaps in prescribing and use of CGMs and AIDs for both children and adults with type 1 diabetes. Our hypothesis is that by standardizing the recommendation for prescribing these technologies using BPAs, we can potentially close gaps in disparities in the use of these technologies. This survey should take no more than 10-15 minutes to complete.

**Eligibility (All Responses Required)**  

1. [age] What is your age?
   1. [Numeric Text] [Disqualify if < 18 years]
2. [dx] Have you been diagnosed with T1D or has your child been diagnosed with T1D?
   1. Yes
   2. No [Disqualify]
3. [caregiver] Are you a Caregiver for a child with T1D?
   1. Yes
   2. No
4. [agechild] [Display to Caregivers, i.e., if caregiver=”Yes”] What is your child’s age?
   1. [Numeric Text]
5. [state] Which state do you currently reside in?
   1. Drop down of states /I do not live in the United States [Disqualify if participant is outside of US]

**Demographics**

1. [hba1c] What was your most recent HbA1c?
   1. Less than 7%
   2. Between 7.0% and 7.9%
   3. Between 8.0% and 8.9%
   4. 9% or higher
   5. I do not remember
2. [gender] What is your gender?
   1. Male
   2. Female
   3. Transgender
   4. Non-binary/genderqueer
   5. I prefer to self-identify [Open Text]
   6. I prefer not to answer
3. [ethnicity] How would you best describe your ethnicity?
   1. Hispanic or Latino
   2. Not Hispanic or Latino
   3. I prefer not to answer
4. [race] How would you best describe your race? *Check all that apply.*
   1. American Indian/Alaskan Native
   2. Asian
   3. Black/African-American
   4. Native Hawaiian or Other Pacific Islander
   5. North African/Middle-Eastern
   6. White/Caucasian
   7. Other (Please specify) [Open Text]
   8. I prefer not to answer [Exclusive]
5. [income] What is your current annual household income from all sources? [Drop down]
   1. $0 to $24,999
   2. $25,000 to $34,999
   3. $35,000 to $49,999
   4. $50,000 to $74,999
   5. $75,000 to $99,999
   6. $100,000 to $124,999
   7. $125,000 to $149,999
   8. $150,000 to $174,999
   9. $175,000 to $199,999
   10. $200,000 or more
   11. I don’t know
   12. Prefer not to answer

**Main Survey**

1. [tech] Are you **currently using** any of these technologies to manage your diabetes? Select ALL that apply:
   1. Continuous glucose monitor (CGM)
   2. Automated insulin delivery system (AID) (a pump and CGM system that automates insulin delivery)
   3. No, I am not currently using any of these technologies [Exclusive]
2. [accept] How **acceptable** would it be to you if your diabetes clinician were prompted by a BPA (computer system alert) to discuss CGMs or insulin pumps with you during a visit?

- Strongly Acceptable
- Acceptable
- Neutral
- Unacceptable
- Strongly Unacceptable

1. [helpful] Do you think it would be **helpful** for patients with type 1 diabetes if a BPA (computer system alert) reminded diabetes clinicians to talk about diabetes technologies, so all patients get the same information?
   - Very Helpful
   - Helpful
   - Neutral
   - Not Helpful
   - Not Helpful at All
2. [aasystem] Do you think using prompts by a BPA (computer system alert) to the diabetes clinician would help more patients with type 1 diabetes **use these technologies**?
   - Strongly agree
   - Agree
   - Neutral
   - Disagree
   - Strongly Disagree
3. [benefits] How do you think patients could **benefit** from diabetes clinicians having a standardized BPA (computer system alert) for continuous glucose monitors (CGMs) and Automated Insulin Delivery systems (AIDs)? (Select all that apply)
   - Improved glycemic control (glucose time in range, A1C, etc.)
   - Reduced hypoglycemic events
   - More consistent care within the healthcare system
   - Increased access to technology
   - Reduced burden of managing diabetes
   - No benefits
   - Other (please specify):
4. [concern] Do you have any **concerns** about the implementation of a BPA (computer system alert) for these devices?
   - Yes
   - No
5. [concernyes] [Display if ‘yes’ to previous question] If yes, what are your concerns? (Select all that apply)
   - Too much dependence on computer-generated clinical advice
   - Too much technology focus for diabetes care
   - Higher healthcare cost
   - Too many alerts for healthcare provider
   - Alert may not be correct based on the information in the electronic medical record
   - Not enough focus on individual patient needs
   - Less control for patients in making decisions about their care
   - Other (please specify):
6. [individual] How important is it for you that the BPA process considers **individual patient preferences and needs**?
   - Very important
   - Important
   - Neutral
   - Not very important
   - Not important at all
7. [personal] To what extent do you agree that personal preferences (e.x. cost, aesthetics, lifestyle, comfort, current use of technology) regarding the use of diabetes technologies are **currently considered** in your diabetes care?
   - Strongly agree
   - Agree
   - Neutral
   - Disagree
   - Strongly disagree
8. [revisitCGM] When discussing **Continuous Glucose Monitors (CGMs)** with your diabetes clinician, how strongly do you feel they should continue to revisit the topic if **you initially decline** these technologies?
   - Not at all: The healthcare team should respect my decision and not bring it up again unless I ask.
   - A little: The healthcare team can briefly mention it in future visits but should not be persistent.
   - Moderately: The healthcare team should occasionally bring it up to ensure I’m informed but should not pressure me.
   - Quite a bit: The healthcare team should regularly revisit the topic and encourage me to consider these technologies.
   - Extremely: The healthcare team should strongly encourage me in every visit until I decide to adopt these technologies.
9. [revisitAID] When discussing **Automated Insulin Delivery systems (AIDs)** with your diabetes clinician, how strongly do you feel they should continue to revisit the topic if **you initially decline** these technologies?
   - Not at all: The healthcare team should respect my decision and not bring it up again unless I ask.
   - A little: The healthcare team can briefly mention it in future visits but should not be persistent.
   - Moderately: The healthcare team should occasionally bring it up to ensure I’m informed but should not pressure me.
   - Quite a bit: The healthcare team should regularly revisit the topic and encourage me to consider these technologies.
   - Extremely: The healthcare team should strongly encourage me in every visit until I decide to adopt these technologies.
10. [BPAdecision] If your **diabetes clinician does not think you are suitable** for an Automated Insulin Delivery (AID) system, **but you think you are**, how strongly do you feel that a Best Practice Advisory (BPA) could help facilitate shared decision-making and support the eventual prescribing of your desired technology?
    - Not at all: I do not believe the BPA would facilitate shared decision-making or support prescribing my desired technology.
    - A little: The BPA might slightly help in facilitating shared decision-making, but not significantly.
    - Moderately: The BPA could somewhat help in facilitating shared decision- making and supporting the prescribing of my desired technology.
    - Quite a bit: The BPA would likely help in facilitating shared decision-making and support prescribing my desired technology.
    - Extremely: The BPA would greatly help in facilitating shared decision-making and strongly support the prescribing of my desired technology.
11. [info] As part of the BPA, what type of **information** would you like to receive from your healthcare provider regarding CGMs and AIDs? (Select all that apply)
    - Benefits and risks
    - Advantages and disadvantages of different devices
    - How to use the device
    - Insurance coverage and costs
    - Educational materials
    - Additional resources to learn more about the devices (e.g. websites, referral to diabetes educators, videos, etc.)
    - Other (please specify):
12. [satCGM] How satisfied are you with the **current level of information** you receive from your healthcare provider about **CGMs**?
    - Very satisfied
    - Satisfied
    - Neutral
    - Dissatisfied
    - Very dissatisfied
13. [satAID] How satisfied are you with the **current level of information** you receive from your healthcare provider about **AIDs**?
    - Very satisfied
    - Satisfied
    - Neutral
    - Dissatisfied
    - Very dissatisfied
14. [caregap] BPAs can also lead to automated messages to patients in the electronic health record portal. How helpful would these **direct-to-patient reminders** about the benefits and use of CGMs and AIDs be in increasing CGM and AID use?
    - Very important
    - Important
    - Neutral
    - Not very important
    - Not important at all
15. [freqreminders] How **frequently** do you think direct-to-patient reminders about the benefits and use of CGMs and AIDs should be sent to patients to ensure they are effective without being overwhelming?
    - More frequently than every 3 months
    - Every 3 months
    - Every 6 months
    - Yearly
    - Other (please specify):
16. [reminders] Have you received any **direct-to-patient** **reminders** about your diabetes management in the electronic medical record portal (e.g., eye exam needed, due for lab work, schedule follow-up appointment, refill needed, etc.)?
    - Yes
    - No
17. [alerthelp] If yes, to what extent do you find these **direct-to-patient reminders** about your **current diabetes management** to be helpful?
    - Very helpful
    - Helpful
    - Neutral
    - Unhelpful
    - Very unhelpful
18. [alerthelp2] If no, to what extent do you think these **direct-to-patient reminders** about your **current diabetes management** might be helpful?
    - Very helpful
    - Helpful
    - Neutral
    - Unhelpful
    - Very unhelpful

Thank you for your participation! Your feedback is invaluable in helping us improve diabetes care and management

**Electronic Survey Questions (Caregiver) – Patient Registry**

Thank you for participating in this survey. Your feedback will help us develop a Best Practice Advisory (BPA) to standardize prescribing Continuous Glucose Monitors (CGMs) and Automated Insulin Delivery systems (AIDs) for children and adults with type 1 diabetes.

A Best Practice Advisory (BPA) is a type of computer system alert tool used to support clinical decision making in electronic health records. BPAs provide alerts and reminders to healthcare providers to ensure that they are following the latest medical guidelines and standards. Current clinical practice guidelines recommend CGMs for all patients with type 1 diabetes, and AIDs (a pump and CGM system that automates insulin delivery) are strongly recommended for patients who can safely use them. Examples of CGMs include Dexcom G7 and Freestyle Libre 3. Examples of AIDs include Tandem Control IQ, Omnipod 5, Medtronic 780G, and iLet Bionic Pancreas.

Despite the benefits of CGMs and AIDs, there are significant racial gaps in prescribing and use of CGMs and AIDs for both children and adults with type 1 diabetes. Our hypothesis is that by standardizing the recommendation for prescribing these technologies using BPAs, we can potentially close gaps in disparities in the use of these technologies. This survey should take no more than 10-15 minutes to complete.

**Eligibility (All Responses Required)**

1. **[age]** What is your age?
   a. [Numeric Text] [] Entry ____
2. **[dx]** Has your child been diagnosed with T1D?
   a. Yes
   b. No [Disqualify]
3. **[caregiver]** Are you a caregiver for a child with T1D?
   a. Yes
   b. No [Disqualify]
4. **[agechild]** What is your child's age?
   a. [Numeric Text]
5. **Less than 3 years old**
6. **Between 3 and 5 years old**
7. **Between 6 and 12 years old**
8. **Between 13 and 18 years old**
9. **Over age 18 years**
10. **[state]** Which state do you currently reside in?
    a. Drop down of states / I do not live in the United States [Disqualify if outside of US]

**Demographics**

1. **[hba1c]** What was your child’s most recent HbA1c?

Less than 7%

Between 7.0% and 7.9%

Between 8.0% and 8.9%

9% or higher

I do not remember

1. How long has your child had Type 1 Diabetes?
2. Less than 1 year
3. 1 to 5 years
4. 6 to 10 years
5. Greater than 10 years
6. I do not remember
7. **[gender]** What is your child's gender?
   a. Male
   b. Female
   c. Transgender
   d. Non-binary/genderqueer
   e. I prefer to self-identify [Open Text]
   f. I prefer not to answer
8. **[race]** How would you best describe your child's race? Check all that apply.
   a. American Indian/Alaskan Native
   b. Asian
   c. Black/African-American
   d. Native Hawaiian or Other Pacific Islander
   e. North African/Middle-Eastern
   f. White/Caucasian
   g. Other (Please specify) [Open Text]
   h. I prefer not to answer [Exclusive]
9. **[ethnicity]** How would you best describe your child’s ethnicity?
   a. Hispanic or Latino
   b. Not Hispanic or Latino
   c. I prefer not to answer
10. **[income]** What is your current annual household income from all sources?
    a. $0 to $24,999
    b. $25,000 to $34,999
    c. $35,000 to $49,999
    d. $50,000 to $74,999
    e. $75,000 to $99,999
    f. $100,000 to $124,999
    g. $125,000 to $149,999
    h. $150,000 to $174,999
    i. $175,000 to $199,999
    j. $200,000 or more
    k. I don’t know
    l. Prefer not to answer

**Main Survey**

1. **[tech]** Is your child **currently using** any of these technologies to manage their diabetes? Select ALL that apply:
   a. Continuous glucose monitor (CGM)

b. Automated insulin delivery system (AID) (a pump and CGM system that automates insulin delivery)

c. No, I am not currently using any of these technologies [Exclusive]

1. **[accept]** How **acceptable** would it be to you if your child’s diabetes clinician were prompted by a BPA (computer system alert) to discuss CGMs or insulin pumps with you during a visit?
   • Strongly Acceptable
   • Acceptable
   • Neutral
   • Unacceptable
   • Strongly Unacceptable
2. **[helpful]** How **helpful** do you think it would be for patients with Type 1 Diabetes if a BPA (computer system alert) reminded diabetes clinicians to talk about diabetes technologies, so all patients get the same information?
   • Very Helpful
   • Helpful
   • Neutral
   • Not Helpful
   • Not Helpful at All
3. **[aasystem]** Do you think using prompts by a BPA (computer system alert) to the diabetes clinician would help more patients with Type 1 Diabetes **use these technologies**?
   • Strongly agree
   • Agree
   • Neutral
   • Disagree
   • Strongly Disagree
4. **[benefits]** How do you think patients could **benefit** from diabetes clinicians having a standardized BPA (computer system alert) for continuous glucose monitors (CGMs) and Automated Insulin Delivery systems (AIDs)? (Select all that apply)
   • Improved glycemic control (glucose time in range, A1C, etc.)
   • Reduced hypoglycemic events
   • More consistent care within the healthcare system
   • Increased access to technology
   • Reduced burden of managing diabetes

Reduced clinician bias in offering diabetes technologies
• No benefits
• Other (please specify):

1. **[concern]** Do you have any **concerns** about the implementation of a BPA (computer system alert) for these devices?
   • Yes
   • No
2. **[concernyes]** [Display if ‘yes’ to previous question] If yes, what are your concerns? (Select all that apply)
   • Too much dependence on computer-generated clinical advice
   • Too much technology focus for diabetes care
   • Higher healthcare cost
   • Too many alerts for diabetes clinician
   • Alert may not be correct based on the information in the electronic medical record
   • Not enough focus on individual patient needs

Feeling pressure to use the diabetes technology
• Less control for patients in making decisions about their care
• Other (please specify):

1. **[individual]** How important is it for you that the BPA process **considers individual patient preferences** (e.g., cost of devices, how it looks on my body, lifestyle considerations, comfort with technology, current use of technology)?
   • Very important
   • Important
   • Neutral
   • Not very important
   • Not important at all
2. **[personal]** To what extent do you agree that **personal preferences** (e.g., cost of devices, how it looks on my body, lifestyle considerations, comfort with technology, current use of technology) regarding the use of diabetes technologies are currently considered in your child's diabetes care?
   • Strongly agree
   • Agree
   • Neutral
   • Disagree
   • Strongly disagree
3. **[revisitCGM]** When discussing **Continuous Glucose Monitors (CGMs)** with your child's diabetes clinician, how strongly do you feel they should continue to revisit the topic if **you initially decline** these technologies?
   • Not at all: The healthcare team should respect my decision and not bring it up again unless I ask.
   • A little: The healthcare team can briefly mention it in future visits but should not be persistent.
   • Moderately: The healthcare team should occasionally bring it up to ensure I’m informed but should not pressure me.
   • Quite a bit: The healthcare team should regularly revisit the topic and encourage me to consider these technologies.
   • Extremely: The healthcare team should strongly encourage me in every visit until I decide to adopt these technologies.
4. **[revisitAID]** When discussing **Automated Insulin Delivery systems (AIDs)** with your child's diabetes clinician, how strongly do you feel they should continue to revisit the topic **if you initially decline** these technologies?
   • Not at all: The healthcare team should respect my decision and not bring it up again unless I ask.
   • A little: The healthcare team can briefly mention it in future visits but should not be persistent.
   • Moderately: The healthcare team should occasionally bring it up to ensure I’m informed but should not pressure me.
   • Quite a bit: The healthcare team should regularly revisit the topic and encourage me to consider these technologies.
   • Extremely: The healthcare team should strongly encourage me in every visit until I decide to adopt these technologies.
5. **[BPAdecision]** If your child’s **diabetes clinician does not think your child is suitable** for diabetes technology, but **you think your child is**, how much do you think a Best Practice Advisory (BPA) could help you and the diabetes clinician make a decision together to eventually start the diabetes technology?
   • Not at all: I do not believe the BPA would facilitate shared decision-making or support prescribing my desired technology.
   • A little: The BPA might slightly help in facilitating shared decision-making, but not significantly.
   • Moderately: The BPA could somewhat help in facilitating shared decision-making and supporting the prescribing of my desired technology.
   • Quite a bit: The BPA would likely help in facilitating shared decision-making and support prescribing my desired technology.
   • Extremely: The BPA would greatly help in facilitating shared decision-making and strongly support the prescribing of my desired technology.
6. **[info]** As part of the BPA, what type of **information** would you like to receive from your child’s diabetes clinician regarding CGMs and AIDs? (Select all that apply)
   • Benefits and risks
   • Advantages and disadvantages of different devices
   • How to use the device
   • Insurance coverage and costs
   • Educational materials
   • Additional resources to learn more about the devices (e.g., websites, referral to diabetes educators, videos, etc.)
   • Other (please specify):
7. **[satCGM]** How satisfied are you with the **current level of information** you receive from your child’s diabetes clinician about **CGMs**?
   • Very satisfied
   • Satisfied
   • Neutral
   • Dissatisfied
   • Very dissatisfied
8. **[satAID]** How satisfied are you with the **current level of information** you receive from your child’s diabetes clinician about **AIDs**?
   • Very satisfied
   • Satisfied
   • Neutral
   • Dissatisfied
   • Very dissatisfied
9. **[caregap]** BPAs can also lead to automated messages to patients in the electronic health record portal. How helpful would these **direct-to-patient reminders** about the benefits and use of CGMs and AIDs be in increasing CGM and AID use?
   • Very helpful
   • Helpful
   • Neutral
   • Not very helpful
   • Not helpful at all
10. **[freqreminders]** How **frequently** do you think direct-to-patient reminders about the benefits and use of CGMs and AIDs should be sent to patients to ensure they are helpful without being overwhelming?
    • More frequently than every 3 months
    • Every 3 months
    • Every 6 months
    • Yearly
    • Other (please specify):
11. **[reminders]** Have you received any **direct-to-patient reminders** about your child’s diabetes management in the electronic medical record portal (e.g., eye exam needed, due for lab work, schedule follow-up appointment, refill needed, etc.)?
    • Yes
    • No
12. **[alerthelp]** If yes, to what extent do you find these **direct-to-patient reminders** about your child’s **current diabetes management** to be helpful?
    • Very helpful
    • Helpful
    • Neutral
    • Unhelpful
    • Very unhelpful
13. **[alerthelp2]** If no, to what extent do you think these **direct-to-patient reminders** about your child’s **current diabetes management** might be helpful?
    • Very helpful
    • Helpful
    • Neutral
    • Unhelpful
    • Very unhelpful

Thank you for your participation! Your feedback is invaluable in helping us improve diabetes care and management.

Top of Form

Bottom of Form
