# Supplementary material for: Implementation and Evaluation of a Best Practice Advisory to Reduce Inequities in Technology Use for People With Type 1 Diabetes: Protocol for a Mixed Methods, Nonrandomized Controlled Trial
Source: JMIR Res Protoc. 2025 May 28;14:e71038. doi: 10.2196/71038 (PMC12159554; doi:10.2196/71038)
Supplement: Multimedia Appendix 5 [file resprot_v14i1e71038_app5.docx]

Implementing Best Practice Advisories to Reduce Inequities in Technology Use for People with Type 1 Diabetes

[When participants arrive in the Zoom room, the moderator will take attendance, ask participants to choose a fake name for the group, and ask them to rename themselves to protect their identity.]

# **Introductory Script**

Good morning/afternoon, my name is XXX, and I am a (insert role) for T1D Exchange. We are inviting adults with Type 1 Diabetes and caregivers to provide input about the Best Practice Advisories (BPA) to reduce inequities in technology use for people with Type 1 Diabetes. The aim is to explore your feedback to standardize the approach for prescribing and documenting advanced diabetes technologies (ADT) (CGM, insulin pump, AID) among PwT1D.

This interview/group should take roughly 45-60 minutes, and it is voluntary. We will ask to record and transcribe it to capture complete responses. Your name will not be attributed to your responses and will be kept confidential. Responses will be aggregated and presented using pseudonyms. Upon completion, you will receive $100 per person for your participation.

When all the interviews/groups are complete, we will use this information to develop the BPA and produce publications and presentations. In addition, T1DX-QI will create a Change Package to guide other centers to replicate these BPA processes. This is your opportunity to have a voice in that process.

Now, I would like to quickly review the group rules. Please speak one at a time and do not share what others say outside this room. Each time you speak, please start with “This is Julia (your fake name)” to help the transcriptionist identify you.

Do you have any other questions before we get started? Do I have your permission to record the interview?

# **Interview Guide**

## Background

- 1. Briefly describe your experience with managing diabetes using advanced technologies like CGMs, insulin pumps, or AID systems?
     - 1. How did you decide to start using (or not use) specific diabetes technology?
       2. How long have you been using a specific diabetes technology?

## Individual Values (Stakeholder Feedback on Patient-Centric Values)

### What considerations are most important to you when deciding whether to use a device like a CGM, insulin pump, or automated insulin delivery (AID) system?

- - - 1. What features like accuracy, ease of use, comfort, or safety do you prioritize when choosing a device?
      2. Are there specific aspects of using these devices (e.g., cost, body image concerns, alarm fatigue) that make them difficult for you to adopt or continue using?
      3. What aspects of your daily routine or activities make certain technologies more or less appealing to you?
      4. Are there specific values, such as independence or privacy, that shape your decision to use (or not use) certain diabetes technologies?
      5. What roles do social situations or interactions play in your willingness to adopt wearable devices like CGMs or insulin pumps?

## Importance of Race (Addressing Equity in BPA Development)

### What role do you think your racial or cultural background has played in the type of care or recommendations you’ve received for managing Type 1 Diabetes?

- - - 1. How has your racial or cultural background shaped the level of trust or understanding you feel with your care team?
      2. Describe any experiences where you felt bias or assumptions by your provider or care team played a role in the recommendations you received for diabetes technology?

### What challenges have you encountered when trying to access diabetes technologies **that you believe are tied to your racial or cultural identity?**

- - - 1. Share any experiences you’ve had with insurance coverage or approvals for diabetes technologies that relate to your racial or cultural identity.
      2. What challenges, like financial or community issues (e.g., traveling long distances to get prescriptions filled, accessing a diabetes specialist, or training on how to use devices like CGMs or insulin pumps) have made it harder for you to access diabetes technologies?
      3. How have healthcare providers’ assumptions about your financial situation affected your ability to access diabetes technologies?
      4. What challenges have you encountered in finding pharmacies, vendors, or specialists that align with your needs in your community?

1. Can you share any specific examples where your race or cultural background may have played a role in the outreach or educational resources you’ve received about managing Type 1 Diabetes?"

### What information or support from providers would help you feel more comfortable with adopting advanced diabetes technologies?

### What steps do you think providers and healthcare systems can take to ensure fair and unbiased recommendations are made for all PwT1D?

### What would help ensure that all patients, regardless of race or cultural background, feel that their needs and preferences are being considered in treatment recommendations?

### How could healthcare providers or systems better support you in overcoming logistical challenges to access diabetes technologies?

### How might the healthcare system improve its processes to make accessing diabetes technology easier for patients like you?

# Feedback on BPA Development

1. What recommendations do you have for improving the way healthcare providers prescribe and support the use of diabetes technologies?
2. How can BPAs be designed to address systemic barriers and ensure equitable care?
   1. What features or processes should BPAs include to ensure they address systemic barriers to diabetes technology?
   2. How can BPAs be designed to promote fair and unbiased recommendations for diabetes technologies?
3. What role should patient and caregiver input play in the development of BPAs?
4. What advice would you give to healthcare providers or systems working to improve diabetes technology access and support?
5. Is there anything else that you would like to tell us or anything important that we have missed?

## Conclusion

I want to thank you for participating in the interview today and for sharing your experiences and perceptions. The information you have provided will be very helpful in informing the BPA to reduce inequities in technology use for people with Type 1 Diabetes.
