# Supplementary material for: Implementation and Evaluation of a Best Practice Advisory to Reduce Inequities in Technology Use for People With Type 1 Diabetes: Protocol for a Mixed Methods, Nonrandomized Controlled Trial
Source: JMIR Res Protoc. 2025 May 28;14:e71038. doi: 10.2196/71038 (PMC12159554; doi:10.2196/71038)
Supplement: Multimedia Appendix 6 [file resprot_v14i1e71038_app6.docx]

**Prompt Submitted to ChatGPT for Figure Generation:**

I have an Excel file with the timeline of my research project. It contains a list of study activities, with corresponding start and end months, and a column indicating whether each activity falls under "Phase 1: Implementation" or "Phase 2: Evaluation."

Please generate a Gantt chart (horizontal bar chart) to visually represent the study timeline. Each bar should span from the start to the end month for each activity. Activities should be listed along the Y-axis in reverse order of appearance in the Excel file (so the first activity appears at the top).

Color-code the bars by phase:

- Blue for "Phase 1: Implementation"

- Green for "Phase 2: Evaluation"

Include a legend for the phases, and label the X-axis as “Timeline (in Months)” and title the chart “Study Timeline.” Make sure the time axis is labeled in 3-month increments from Month 0 to Month 27.

Use matplotlib in Python to generate the figure, and use `plt.tight_layout()` to ensure everything fits well.

**Prompt for TOC figure generation.**

Create a stylized illustration suitable for the Table of Contents (TOC) of a scientific journal article. The image should represent the use of a Best Practice Advisory (BPA) within an electronic health record (EHR) system to promote health equity in diabetes technology use.

On the left side of the image, show a desktop computer screen displaying a warning or alert symbol (such as a red exclamation mark inside a triangle) along with a pop-up notification or alert window. This represents the BPA.

On the right side of the image, show a continuous glucose monitor (CGM) and an insulin pump (AID system), including a wireless signal icon to indicate real-time data transmission.

Use a clean, flat, minimalistic design with soft colors and outlines, appropriate for an academic publication. The background should be neutral or light to avoid distraction.

**Do not include any text or title in the image.** The figure should focus solely on the visual elements.
